# How Concerted Are Ionic Hops in Inorganic Solid-State Electrolytes?

**DOI:** 10.1021/jacs.3c13279

**Published:** 2024-03-18

**Authors:** Cibrán López, Riccardo Rurali, Claudio Cazorla

**Affiliations:** † Departament de Física, Universitat Politècnica de Catalunya, 08034 Barcelona, Spain; ‡ Barcelona Research Center in Multiscale Science and Engineering, Universitat Politècnica de Catalunya, 08019 Barcelona, Spain; § Institut de Ciència de Materials de Barcelona, ICMAB−CSIC, Campus UAB, 08193 Bellaterra, Spain

## Abstract

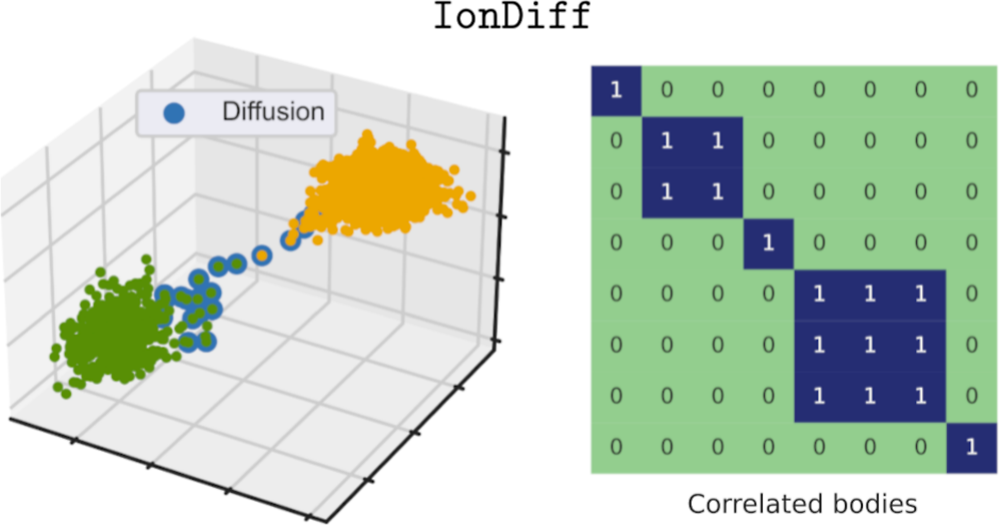

Despite being fundamental to the understanding of solid-state electrolytes (SSEs), little is known on the degree of coordination between mobile ions in diffusive events, thus hindering a detailed comprehension and possible rational design of SSEs. Here, we introduce an unsupervised k-means clustering approach that is able to identify ion-hopping events and correlations between many mobile ions and apply it to a comprehensive ab initio MD database comprising several families of inorganic SSEs and millions of ionic configurations. It is found that despite two-body interactions between mobile ions being the largest, higher-order *n*-ion (2 < *n*) correlations are most frequent. Specifically, we prove a general exponential decaying law for the probability density function governing the number of concerted mobile ions. For the particular case of Li-based SSEs, it is shown that the average number of correlated mobile ions amounts to 10 ± 5 and that this result is practically independent of the temperature. Interestingly, our data-driven analysis reveals that fast-ionic diffusion strongly and positively correlates with ample hopping lengths and long hopping spans but not with high hopping frequencies and short interstitial residence times. Finally, it is shown that neglection of many-ion correlations generally leads to a modest overestimation of the hopping frequency that roughly is proportional to the average number of correlated mobile ions.

## 1 Introduction

Solid-state electrolytes (SSEs) presenting high ionic conductivity are pivotal for the development of transformative green-energy conversion and storage technologies like fuel cells, electrocatalysts, and solid-state batteries.^1−4^ SSEs are complex materials that exhibit very disparate compositions, structures, thermal behaviors, and ionic mobilities; hence, unfortunately, it is difficult to rationally ascribe them to general categories and design principles.^5,6^ In particular, there is a lack of fundamental knowledge about the collective atomistic mechanisms that govern ionic transport.

In recent years, analysis of the correlations between ionic transport (i.e., mobile ions) and lattice dynamics (i.e., vibrating ions) have attracted increasing interest.^6−9^ The “paddle-wheel” mechanism, in which the libration of semirigid anionic units can propel cation transport,^10^ is a well-known example of such a possible type of atomic concertation in ionic conductors. The influence of lattice anharmonicity on ionic transport has been also thoroughly discussed, both theoretically and experimentally.^11−14^ Nonetheless, very little is known about the existing level of coordination between many mobile ions in diffusive events.

Thus far, identification of correlations between mobile ions mostly has relied on the analysis of van Hove correlation functions obtained from ab initio molecular dynamics (AIMD) simulations and on zero-temperature nudged elastic band (NEB) calculations.^15−17^ For Li-based SSEs, it has been theoretically demonstrated that concertation between many mobile ions tends to lower the energy barriers for ionic diffusion, hence collective diffusive behavior, rather than individual ionic hops, is expected to be predominant in fast-ionic conductors.^17^

Nevertheless, due to the inherent limitations of the analysis methods employed thus far (e.g., correlations beyond two bodies cannot be quantified with van Hove functions, and temperature effects are disregarded in NEB calculations), many questions on the level of concertation between many mobile ions remain unanswered. For example, how many ions are typically coordinated in diffusive events and through which collective mechanisms? Are these many-ion correlations dependent on temperature or not? Can collective hopping behavior be analytically described by a general law? Does the degree of ionic coordination depend on the specific SSE family, or is it general? How does the neglection of many-ion correlation affect the estimation of key atomistic quantities like the ion hopping frequency? Answering these questions is not only relevant from a fundamental point of view but also necessary to justify the broad adoption of formulas obtained in the dilute-solution limit (e.g., the Nerst–Einstein relation for the ionic conductivity), which assumes mobile ions to be fully uncorrelated.^18−22^

In this work, we introduce a k-means clustering approach that is able to unsupervisedly identify ion-hopping events and quantify correlations between many mobile ions from ionic configurations generated in atomistic molecular dynamics simulations. This automatized analysis was recursively applied on a comprehensive AIMD database comprising several families of inorganic SSEs and millions of atomic configurations.^6,24^ It was found that many-ion correlations beyond pairwise are dominant in diffusive events and can be represented by a general exponential decaying law. Interestingly, for Li-based SSEs, it was determined that the average number of concerted mobile ions amounts to 10 ± 5, very much independently of the temperature. Moreover, the introduced unsupervised analysis also permitted us to accurately quantify the prevalent correlations between ionic diffusion and key microscopic quantities such as ion hopping lengths and frequencies and interstitial residence times. In addition, the effects of neglecting many-ion correlations on the estimation of the ion hopping frequency and migration energy barrier were substantiated. Therefore, the present work leverages our fundamental understanding of technologically relevant SSEs and elaborates on the adequacy of employing formulas obtained within the dilute-solution limit for describing them.

## 2 Results

**Figure 1:**
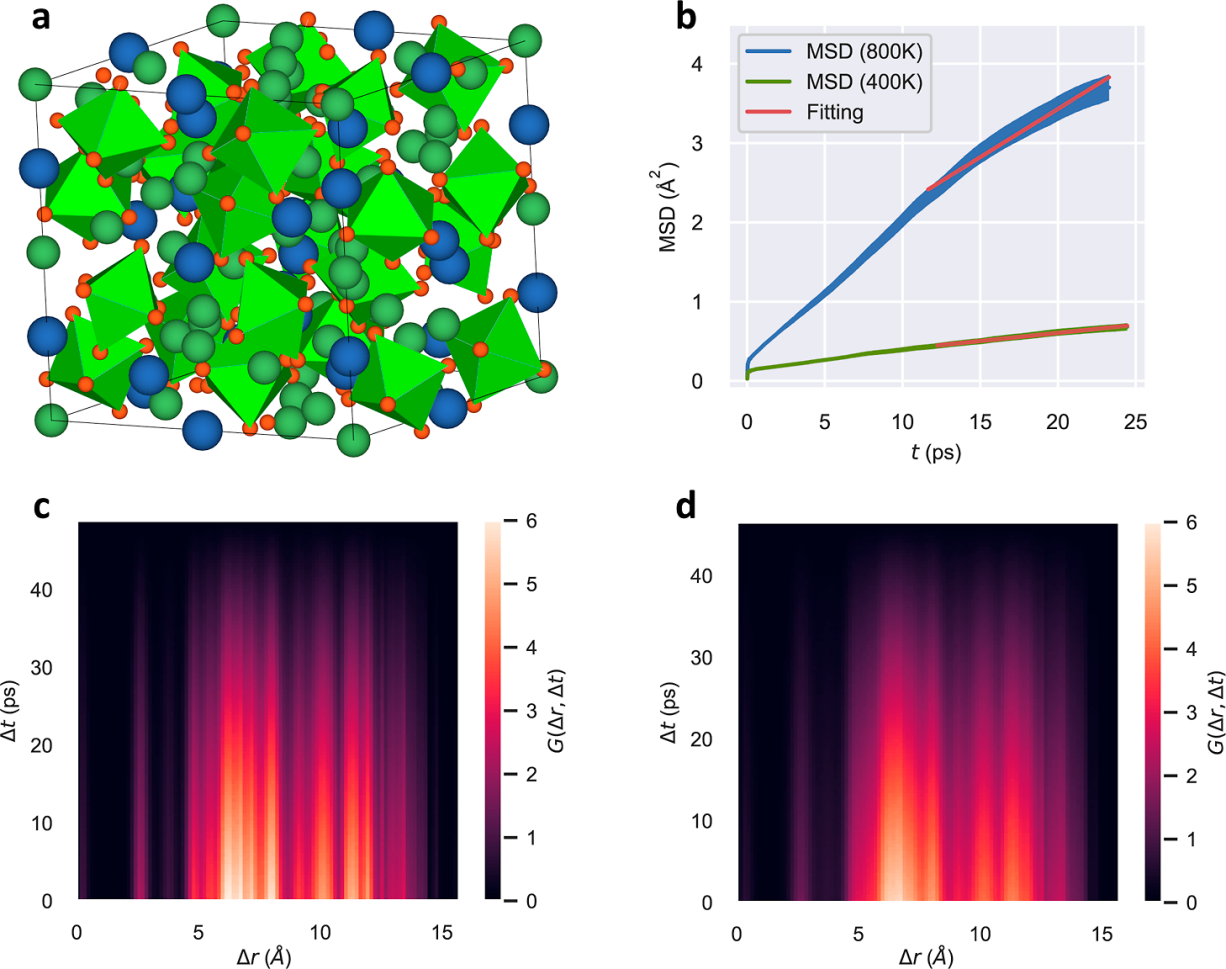
Standard characterization of ionic transport and correlations in SSE from molecular dynamics simulations particularized for Li_7_La_3_Zr_2_O_12_ (LLZO). (a) Ball-stick representation of bulk LLZO presenting a low-temperature tetragonal garnet-like crystal structure (space group *I*4_1_/*acd*); lanthanum, lithium, oxygen, and zirconium atoms are represented with dark blue, green, red, and light blue spheres, respectively. (b) MSD of Li cations obtained from DFT-AIMD simulations performed at *T* = 400 and 800 K and considering the low-temperature tetragonal garnet-like structure. (c,d) van Hove correlation function for Li cations (in arbitrary units) obtained from DFT-AIMD simulations performed at *T* = 400 and 800 K, respectively.

Figure 1 shows the results of finite-temperature AIMD simulations performed for Li_7_La_3_Zr_2_O_12_ (LLZO), an archetypal Li-based SSE.^23^ LLZO is a complex oxide material that at temperatures below ≈600 K stabilizes in a tetragonal garnet-like structure (space group *I*4_1_/*acd*, Figure 1a) with a moderate lithium-ion conductivity of ∼10^–6^ S cm^–1^ and excellent thermal and chemical stabilities; at higher temperatures, LLZO transforms into a cubic phase that presents a considerably higher Li conductivity (∼10^–4^ S cm^–1^).^25−28^ As it is customarily done for SSEs, one can estimate the tracer Li diffusion coefficient of LLZO, *D*_Li_, directly from the configurations generated during AIMD simulations by computing the time derivative of the corresponding mean squared displacement (MSD) (Figure 1b and Computational Methods).^8,29^ Larger *D*_Li_ values are associated with larger ionic conductivities, σ_Li_, as deduced from the popular Nernst–Einstein relation obtained in the dilute-solution limit
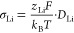
1where *z*_Li_ represents the charge of the mobile ion, *k*_B_ is the Boltzmann constant, and *F* = *e* · *N*_A_ is the Faraday constant (*e* is the electron charge and *N*_A_ is Avogadro’s number).

The van Hove correlation function, *G*(Δ*r*, Δ*t*) (Computational Methods), provides information on the spatiotemporal distribution of pairs of particles in atomistic configurations obtained from finite-temperature MD simulations (e.g., for a null time span, *G* is equivalent to the usual radial pair distribution function). Figure 1c,d shows the van Hove correlation function of Li atoms estimated for the ionic conductor LLZO at two different temperatures; it is appreciated that pair correlations between nearby ions, that is, 2 ≤ Δ*r* ≤ 5 Å, are substantial over time spans of several tens of picoseconds since *G*(Δ*r*, Δ*t*) remains discernible within those intervals. At the highest simulated temperature, ionic diffusion is sizable (Figure 1b), and the peaks of the van Hove correlation function (Figure 1d) get noticeably faded (barely change) along the interparticle distance (time) dimension in comparison to those obtained for the nonconductive state (Figure 1c). For completeness purposes, the “self” and “distinct” components of the lithium van Hove correlation function (Computational Methods) are shown in Supporting Figure 1. These *G*(Δ*r*, Δ*t*) results clearly show the existence of significant ion-pair correlations in LLZO ionic diffusion.

Nevertheless, the standard particle correlation analysis presented above is too restricted since it only considers correlations between pairs of atoms, thus neglecting any possible higher-order level of *n*-ion (2 < *n*) concertation. In addition, it does not provide any atomistic insight into the many-ion mechanisms involved in ionic diffusion. To overcome this type of limitation, we devised an algorithm based on k-means clustering that is able to unsupervisedly identify ion-hopping events and correlations between many particles and applied it to a comprehensive AIMD database of inorganic SSEs.^6,24^ The introduced algorithm also permits the automatic identification of ion hopping lengths and frequencies and interstitial residence times; hence, the general dependencies between these atomistic descriptors and ionic diffusion can be determined.

### 2.1 K-Means Clustering Algorithm for Unsupervised Identification of Ionic Hops and Diffusive Paths

Our approach consists in identifying the equilibrium and metastable positions in a supercell around which particles vibrate considering periodic boundary conditions; subsequently, the temporal sequence of atomic displacements from one of those vibrational centers to another is monitored, thus determining ion diffusion paths without imposing any restriction. Only two fundamental premises are assumed in our procedure; namely, the vibration of ions around equilibrium and metastable positions are roughly isotropic, and diffusion events are less frequent than atomic vibrations.

K-means clustering is an unsupervised machine learning algorithm that classifies objects in such a way that elements within the same group, called “cluster”, are in a broad sense more similar to each other than to elements in other clusters. Our method for identifying vibrational centers from sequential ionic configurations relies on k-means clustering (Computational Methods) since this approach assumes isotropy on the fluctuations of nondiffusive particles. It is worth mentioning that spectral clustering, based on interparticle connectivity instead of interparticle distance, was also considered; however, less satisfactory ionic hop identification results were obtained in this case. Importantly, the definition of arbitrary material-dependent threshold distances for scrutiny of ionic hops is completely avoided in our approach, as we explain next.

For each individual ionic trajectory, the optimal number of clusters, *K*, which represents the number of vibrational centers that the particle visits during the simulation, is systematically selected as the one that maximizes the silhouette coefficient averaged over all the samples corresponding to cases 2 ≤ *K* (Computational Methods). Silhouette coefficients, *S*, are individually ascribed to each cluster and can take values within the interval [−1, +1]. *S* values near +1 indicate that the sample is far away from the neighboring clusters. On the other hand, negative *S* values indicate that the sample might has been assigned the wrong cluster (an exact zero value would indicate that the sample is on the decision boundary between two neighboring clusters). Nevertheless, this procedure fails to describe the case of a nondiffusive particle, which would correspond to *K* = 1, since by construction 2 ≤ *K*. To avoid this issue, whenever the maximum average silhouette coefficient is below an arbitrary but reasonable threshold value of 0.7, we automatically impose *K* = 1 (i.e., the ion does not diffuse throughout the simulation). The dependence of our algorithm performance on such a threshold value has been exhaustively tested, finding negligible effects on the final outcomes.

Once the number of vibrational centers, their real-space location, and temporal evolution are determined, ionic diffusion paths are defined as the fragments connecting two different vibrational centers through time. Due to the discrete nature of the generated trajectories and technicalities of the k-means clustering approach, it is difficult to unequivocally establish the start and end points of ionic diffusion paths; thus, an arbitrary but physically reasonable threshold distance of 0.5 Å from the midpoint of the vibrational centers has been adopted here to define the extremities of diffusive trajectories. It is noted that a similar, although not identical, k-means clustering algorithm for unsupervised identification of ionic hops was recently developed by others and applied to the study of an oxide solid electrolyte.^30^

**Figure 2:**
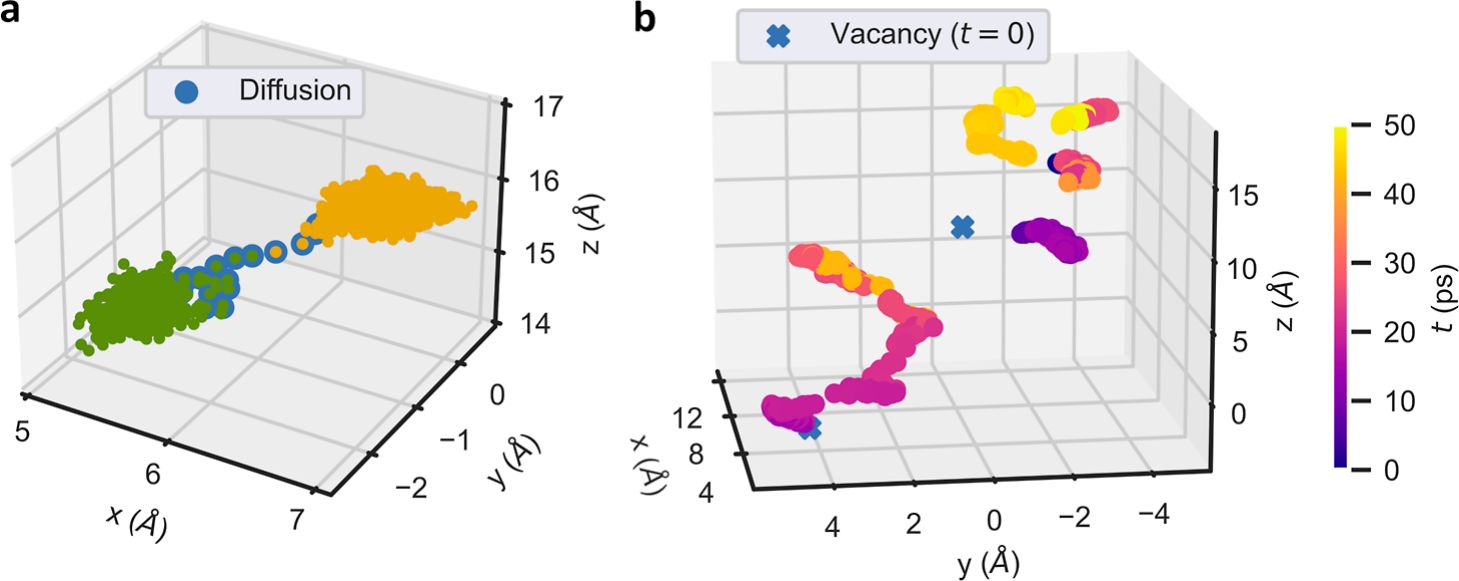
Unsupervised k-means clustering algorithm for identification of ionic hops and diffusion paths. (a) Ionic diffusion of an arbitrary mobile atom in a DFT-AIMD simulation of LLZO performed at *T* = 400 K (blue circles). The two vibration centers defining the origin and end of the ionic hop are represented with orange and green points, respectively. (b) Temporal sequence of ionic hops identified for a ≈50 ps duration of DFT-AIMD simulation of LLZO performed at *T* = 400 K. Blue crosses represent the initial position of two lithium vacancies introduced in the simulation cell; ionic hops are initiated near them. Different sections of a same diffusion path do not necessarily correspond to a same ion.

An illustrative example of our method for identification of vibrational centers and ionic diffusion paths is shown in Figure 2a. Therein, two vibrational centers with a highly confident average silhouette coefficient value of 0.88 (green and yellow points) are depicted along with the ionic diffusion path (blue points) that connects them. Our algorithm was recursively applied to a comprehensive DFT-AIMD database involving different families of SSEs^6,24^ (Supporting Tables I–III), obtaining in all the cases highly accurate results for the identification of ionic hops and diffusive paths. For example, for nonstoichiometric LLZO (i.e., containing Li vacancies), simulated at temperatures of 400 and 800 K, reassuring average silhouette coefficients amounting to 0.99 and 0.97 were, respectively, obtained (Figure 2b).

It is worth noting that our ionic hop identification algorithm fully takes into account periodic boundary conditions and neither presupposes a fixed number nor the positions of vibrational centers in the provided atomistic configurations (e.g., the number of vibrational centers may differ from the number of potentially mobile atoms when there is significant ionic diffusion, Supporting Figure 2). This adaptability feature turns out to be particularly useful for the identification of metastable crystalline positions (e.g., interstitials) and evaluation of residence times, as shown later. The explained analysis method has been implemented in the IonDiff software,^31^ a free open-source python code (Computational Methods).

### 2.2 Quantitative Analysis of Concertation between Many Mobile Ions

The ionic hop identification approach explained above was practiced on a comprehensive DFT database of inorganic SSEs comprising a total of 61 materials, of which 46% contain Li, 23% halides (F, Cl, Br, and I), 15% Na, 8% O, and 8% Ag/Cu atoms as the mobile ions.^6,24^ These percentages were originally selected to roughly reproduce the relative abundances of fast-ionic conductors reported in the literature.^32^ Since we are primarily interested in unveiling general behaviors and relationships in ionic transport, we ended up applying our formalism on a total of 83 AIMD simulations (Computational Methods) in which ionic diffusion was substantial (Supporting Tables I–III). Neither hybrid organic–inorganic nor one-dimensional ionic conductors (e.g., anionic metal–organic frameworks,^33^ LiCuVO_4_,^34^ and KTiOPO_4_^35^) were included in our analysis. The types of imperfections rendered by the AIMD simulations were Schottky and Frenkel defects for nonstoichiometric and stoichiometric systems, respectively. Further details of the analyzed materials and simulations (e.g., crystal symmetry and supercell sizes) can be found in the Computational Methods section and Supporting Tables I–III.

**Figure 3:**
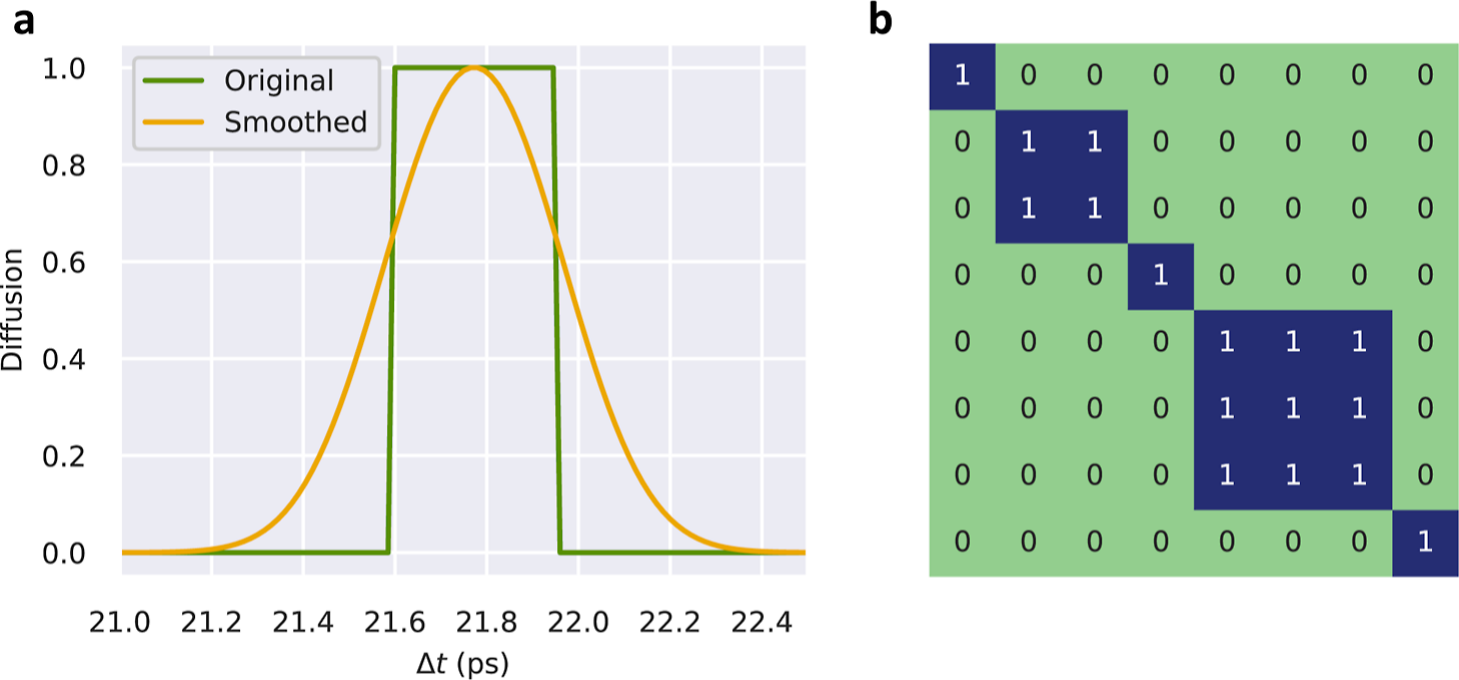
Unsupervised estimation of correlations between many mobile ions. (a) At each time step, the state of each mobile ion is identified with a “0”, if it is vibrating, or a “1”, if it is hopping. The multistep functions obtained over time are smoothed out with Gaussian functions to improve the numerical convergence in the subsequent calculation of the many-ion correlation matrix. (b) Considering all the binary data generated during a molecular dynamics simulation, a *N* × *N* correlation matrix is obtained, *N* being the number of possible mobile ions, which provides the number and indexes of uncorrelated and correlated ions (represented by “0” and “1”, respectively). In the provided example, a group of two ions and another of three move concertedly, while three particles remain uncorrelated during the whole simulation.

To quantitatively evaluate the correlations and level of concentration between an arbitrary number of mobile ions, *n*, we devised and implemented the following algorithm. For a given sequence of ionic configurations generated during a molecular dynamics simulation, the corresponding correlation matrix for diffusive events was computed. To this end, we first assigned a value of “1” to each diffusing particle and a value of “0” to each vibrating particle at each time frame (Figure 3a). Such a binary numerical assignment was straightforwardly performed with the ionic hop identification algorithm introduced in the previous section. Due to the discrete nature of the generated ionic trajectories and to improve numerical convergence in the subsequent correlation analysis, the obtained multistep time functions were approximated with Gaussians that equaled the half maxima at their width (Figure 3a, in analogy to the “full-width-at-half-maximum”—fwhm—method widely employed in signal processing). Subsequently, we computed the *N* × *N* correlation matrix, where *N* is the number of potentially mobile ions, resulting from all the gathered simulation data; this latter step involves the calculation of covariance coefficients for ions taken in pairs.^6^

The correlation matrix thus estimated, however, may be difficult to converge due to its statistical character (particularly in situations where the number of mobile ions and time steps are somewhat limited, as tends to be the case for AIMD simulations). Moreover, eventual uncorrelated ion hops that incidentally occur at the same time could be incorrectly regarded as correlated. To overcome these practical issues, we computed a reference correlation matrix corresponding to a randomly distributed sequence of ionic hops with the Gaussian fwhm equal to the mean diffusion time determined during the simulation (note that due to the finite width of the Gaussians such a correlation matrix is not exactly equal to the identity). Subsequently, covariance coefficients in the original correlation matrix larger (smaller) than the corresponding random reference values were considered as true correlations (random noise) and hence were rounded off to one (zero) for simplification purposes. To not underrate the many-ion correlations, different hops of the same ion were treated as independent events.

In this manner, a correlation matrix consisting of ones and zeros is finally assembled from which one can easily determine how many particles remain concerted during diffusion. Figure 3b shows a correlation matrix example in which a group of two mobile atoms and another of three move concertedly, while three ions remain uncorrelated during the whole simulation (rows and columns have been reshuffled in order to facilitate the visualization of many-ion correlations). The described many-ion correlation identification algorithm also has been implemented in the IonDiff software,^31^ a freely available open-source python code (Computational Methods).

### 2.3 Probability Density Function Governing the Number of Correlated Mobile Ions

**Figure 4:**
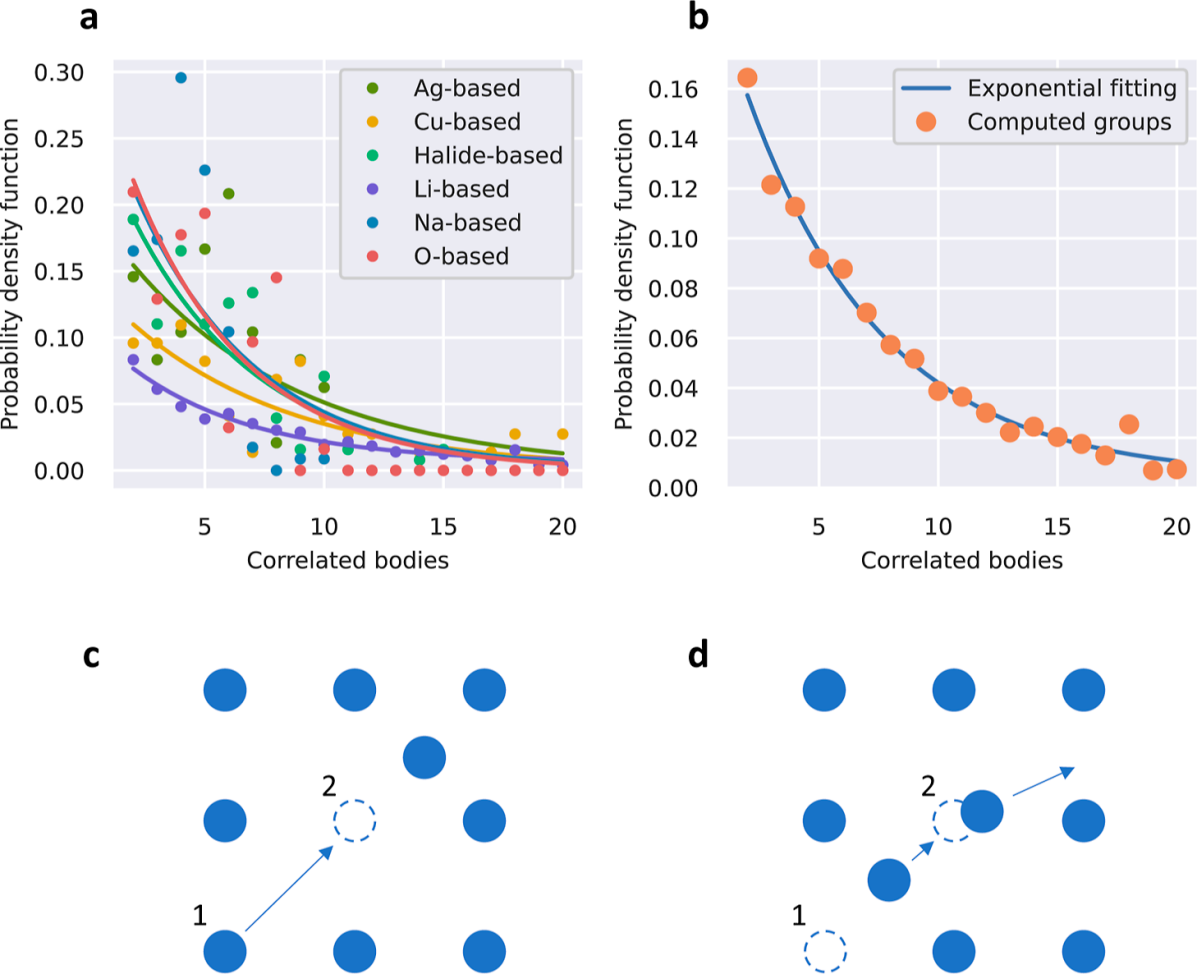
Many-ion correlation results obtained from applying the introduced unsupervised k-means clustering algorithm on a comprehensive DFT-AIMD database of inorganic SSEs.^24^ (a) Probability density function for the number of coordinated ions in collective ionic hops estimated separately for each SSE family. High-order many-ion correlations are most substantial in Cu- and Li-based fast-ionic materials. Solid lines represent exponential decaying fits to the data points. (b) General probability density function for the number of coordinated ions in collective ionic hops estimated considering all SSE compounds. An exponential decaying function of the form *f*(*n*) = 0.220·exp(−0.252*n*) fits fairly well the obtained data points. Two different two-ion-coordinated mechanisms were most frequently observed in diffusive events: (c) ion (1) moves toward an empty equilibrium lattice position just left by ion (2), and (d) a mobile ion (1) kicks out a vibrating atom (2) and occupies its equilibrium lattice position.

Figure 4a shows the probability density function (pdf) that governs the number of concerted ions in diffusive events estimated for different SSE families (i.e., averaged over compounds belonging to the same category and temperature). These results were obtained from AIMD simulations that fully take into account anharmonicity and temperature effects.

In all cases, an exponential decaying function was found to fairly reproduce the estimated distribution of *n*-concerted ions (solid lines in Figure 4a). Consequently, the degree of concertation between mobile particles is always largest for pairs of ions and steadily decreases for an increasing number of ions (here, we arbitrarily but reasonably considered only cases up to *n* = 20). The value of the pre-exponential factor and parameter in the exponential function, however, significantly vary from one family of materials to another. Therefore, the level of many-ion coordination in diffusive events depends on the specific SSE group. In particular, O-, halide-, and Na-based fast-ionic conductors exhibit the most rapidly decaying pdf profiles, meaning that correlations for a large number of mobile ions are smallest. On the other hand, Cu- and Li-based fast-ionic conductors display the most slowly decaying pdf profiles (i.e., correlations for a large number of mobile ions are largest), while Ag-based SSEs show an intermediate trend.

Figure 4b shows the general pdf obtained for the number of concerted mobile ions in fast-ionic conductors (i.e., averaged over all SSE families and temperature). An exponential decaying law is found to reproduce the estimated distribution of *n*-ion correlations. In this general case, the degree of particle concertation is also largest for pairs of ions, as expected. However, by performing integrations of the area enclosed below the solid line in Figure 4b, it is found that coordinated diffusive events involving more than two ions turn out to be more frequent (roughly by a factor of 6). This finding, which follows from comprehensive AIMD simulations and is not restricted to an unique SSE family, is consistent with previous computational results reported for Li-based materials.^17^

Our formalism also allows one to identify which particles participate in the disclosed *n*-ion-coordinated diffusion events, which is very convenient for data visualization purposes. For the special case of *n* = 2 correlated diffusion processes, we determined the two most relevant atomistic coordination mechanisms, which consistently were found to occur in all the analyzed SSE families (sketched in Figure 4c,d). The first mechanism consists in a sequence of two diffusion events in which a first mobile ion hops to an interstitial position leaving a vacant site that is immediately occupied afterward by a second diffusing particle (Figure 4c). The second mechanism consists of the forced jump of a particle resulting from the direct influence of a second diffusing ion (Figure 4d). It is worth noting that these two *n* = 2 ionic correlation mechanisms have been already reported in the literature for Li-based compounds,^36^ thus confirming the reliability of our unsupervised ionic-hop identification approach.

We note that, despite the significant computational effort invested, the current scope and diversity of the investigated SSE database^6,24^ may not be expansive enough to conclusively affirm the universality of the obtained results. For instance, our study has omitted hybrid organic–inorganic materials, one-dimensional ionic conductors, and crystal imperfections beyond Schottky and Frenkel defects, such as ion substitution. However, the considerable heterogeneity of the analyzed materials and ionic diffusion mechanisms provide a certain level of generality to our conclusions. Future research aimed at expanding the repertoire of SSE and analysis of various ionic transport processes using the IonDiff software^31^ could benefit from employing machine learning interatomic potentials, such as universal graph-based force fields (e.g., M3GNet^37^). Nonetheless, it is essential to acknowledge that these approaches may not be devoid of limitations (Supporting Discussion).

### 2.4 Temperature Dependence of Many Mobile Ion Correlations

An interesting question to answer for fast-ionic conductors is whether the degree of concertation between many mobile ions depends on temperature or not.^18,19,38^ The findings reported in the previous section cannot provide direct insights into this question since they were obtained from thermal averages. Consequently, we performed a detailed temperature analysis of the many-ion correlations identified for Li-based compounds alone since these are technologically very relevant and relatively abundant.

**Figure 5:**
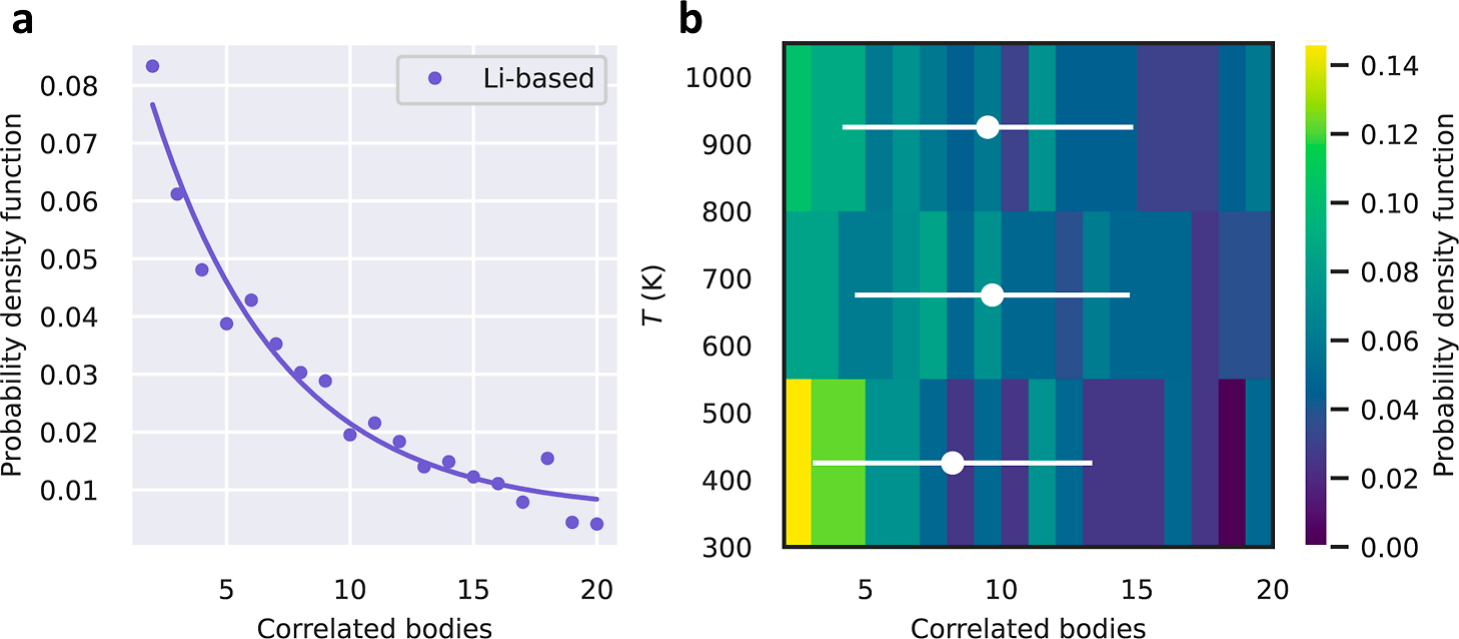
Many-ion correlation results obtained for Li-based SSEs. (a) Probability density function for the number of concerted ions in diffusive events. The solid line represents an exponential decaying fit to the data points. (b) Temperature-dependence of the probability density function shown in (a) considering the intervals 300 ≤ *T*_1_ ≤ 550 K, 550 ≤ *T*_2_ ≤ 800 K, and 800 ≤ *T*_3_ ≤ 1050 K. White dots and lines denote average values and corresponding standard deviations, respectively.

Figure 5a shows the pdf estimated for the number of concerted many mobile ions in Li-based SSEs (same as in Figure 4a). By taking all the collective diffusive events represented in that figure, we constructed normalized temperature histograms considering the three intervals 300 ≤ *T*_1_ ≤ 550 K, 550 ≤ *T*_2_ ≤ 800 K, and 800 ≤ *T*_3_ ≤ 1050 K, as shown in Figure 5b. Very mild differences are appreciated for the pdf’s estimated for such temperature ranges. For example, at low temperatures, coordinated diffusion events involving pairs of ions appear to be more frequent than at high temperatures. However, when average quantities are considered, such moderate discrepancies mostly disappear. Specifically, the average number of coordinated mobile ions approximately amounts to 10 ± 5 for all of the investigated temperature intervals (white dots and lines in Figure 5b). Therefore, we may conclude that the level of concertation between mobile ions in Li-based SSEs is practically independent of temperature.

### 2.5 Relationship between Ionic Diffusion and Key Atomistic Descriptors

As explained in previous sections, the IonDiff software^31^ allows one to determine the centers of vibration and exact migrating paths of ions as provided by molecular dynamics simulations. Accordingly, for a given sequence of ionic configurations, it is straightforward to estimate insightful atomistic descriptors such as the average hopping distance, Δ*r*, hopping time, Δ*t*, and hopping frequency, ν. Likewise, it is also possible to estimate interstitial residence times, γ, by monitoring the simulation time during which a particle remains fluctuating around a metastable position (e.g., interstice). The identification of metastable positions was performed by comparing the centers of vibration obtained during a whole simulation with those of the perfect equilibrium configuration and assuming that metastable and equilibrium vibrational centers should be separated by a distance of at least 1.0 Å (Supporting Figure 3).

**Figure 6:**
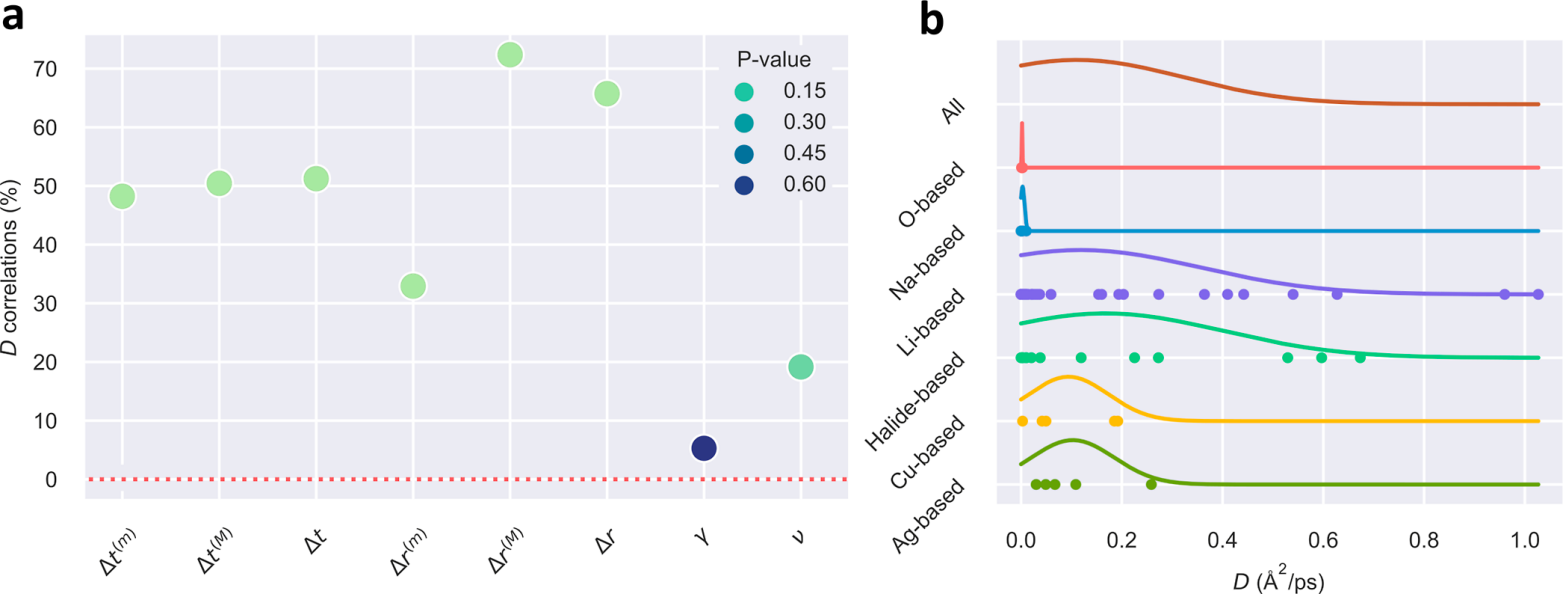
Correlations between ionic diffusion and key atomistic descriptors. (a) *D* stands for the tracer ion diffusion coefficient, Δ*t* is the average duration of an ionic hop, Δ*r* is the average length of an ionic hop, γ is the average interstitial residence time, and ν is the hopping frequency. Superscripts “(M)” and “(m)” denote maximum and minimum values estimated for the corresponding descriptor. (b) Distribution of tracer ion diffusion coefficients calculated for each SSE family.

Figure 6 shows the level of correlation estimated for the tracer ion diffusion coefficient, *D*_*x*_, and atomistic descriptors described above considering all the SSE families examined in this study (for this analysis, we considered the tracer diffusion coefficient instead of the full ion diffusion coefficient^19−21^ because of its ubiquity in computational studies). Such correlations were obtained by following the same data-analysis approach that was introduced in our previous work,^6^ which essentially involves the computation of Spearman correlation coefficients and *p*-values for the assessment of statistical significance. Besides examining average quantities, for the case of Δ*r* and Δ*t*, we also considered their maximum, “(M)”, and minimum, “(m)”, values. Several interesting conclusions follow from the results shown in Figure 6a.

The largest *D*_*x*_ correlations involving average quantities are found for the hopping length and hopping time, which are both positive and roughly amount to 65 and 50%, respectively. In the particular case of Δ*r*, the maximum ion diffusion correlation is obtained for its maximum value, Δ*r*^(M)^, which is above 70% (Figure 6a). On the other hand, the smallest *D*_*x*_ correlation is found for the average interstitial residence time, which only amounts to ≈5%. As for the hopping frequency, the level of correlation with the ion diffusion coefficient is also positive but quite reduced (≈20%). In most cases, the estimated correlations turn out to be statistically significant since the accompanying *p*-values are equal or smaller than 0.10.^6^ For a detailed description of the examined data, Figure 6b shows the distribution of tracer ion diffusion coefficients calculated for each SSE family, which turns out to be quite diverse.

Based on this data-driven atomistic analysis, we may conclude that good ionic conductors characterized by large ion diffusion coefficients should present large hopping lengths and hopping times but not necessarily high hopping frequencies or short interstitial residence times (Figure 6a). To put it differently, ample and timely, rather than short and too frequent, ionic hops appear to be associated with high ionic diffusion.

To gain further insight into the connections between high ionic diffusion and key atomistic descriptors, Supporting Figure 4 shows the *T*-dependence of ν and Δ*r* as evaluated for different SSE families. In general, it is found that the hopping frequency does not appreciably change with temperature, whereas the average hopping distance noticeably increases upon increasing temperature. These results imply that the general *T*-induced ionic diffusion enhancement observed in SSEs mostly is mediated by a surge in Δ*r* rather than in ν. In turn, these findings appear to be coherent with the main conclusion presented in the preceding paragraph, namely, that the influence of the average hopping distance on fast-ionic conduction exceeds that of the hopping frequency.

## 3 Discussion

In the dilute-solution limit, the interactions between mobile ions are regarded as negligible; hence, the full ionic diffusion coefficient reduces to the tracer diffusion coefficient^19−21^ (Computational Methods), and its dependence on temperature can be expressed as^8,18^
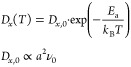
2where *a* is a hopping distance, *E*_a_ is the activation energy barrier for ionic migration, and ν_0_ is the hopping frequency.

The many mobile ion correlation results presented in previous sections show that the dilute-solution limit, in general, does not apply to technologically relevant SSEs; hence, one may question the validity of eq 2 and other commonly employed formulas, like the Nerst–Einstein relation (eq 1 above), obtained under similar approximations. Aimed at quantitatively exploring this objection, we computed the hopping frequencies of all the SSEs analyzed in this study by using eq 2, ν_0_, which assumes the interactions between mobile ions to be negligible, and compared them with the values obtained directly from AIMD simulations with the IonDiff software,^31^ ν, which fully takes into consideration many-ion correlations. Since an undetermined proportionality factor enters eq 2, we constrain our comparative analysis to the orders of magnitude of the examined hopping frequencies.

**Figure 7:**
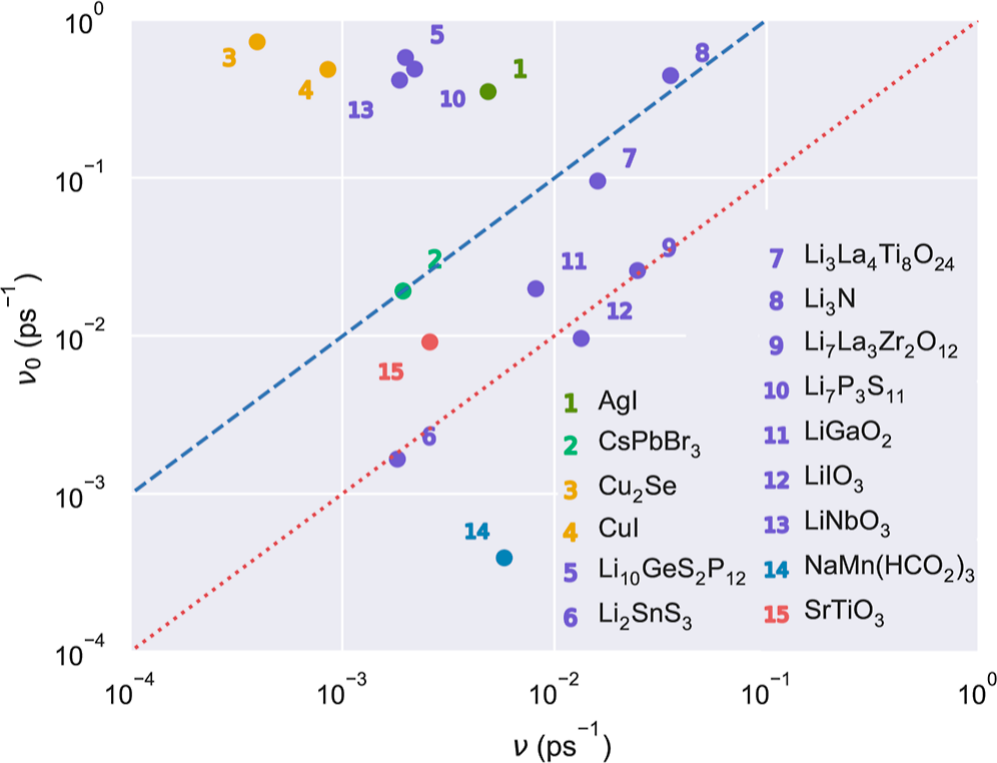
Comparison of the hopping frequencies estimated for representative SSEs in the dilute-solution limit, ν_0_, and by explicitly considering many-ion correlations, ν. The straight lines in the plot indicate the *coincidence* region in which the orders of magnitude of the two represented hopping frequencies coincide or differ to within a factor of 10 while fulfilling the condition ν ≤ ν_0_ (main text).

Figure 7 shows our ν_0_ and ν results obtained for 15 representative fast-ionic conductors. Due to the fact that the proportionality factor entering eq 2 may be of the order of 10^0^–10^1^, we regard as coincidence that a pair of ν_0_–ν hopping frequencies differs within such a quantity and fulfills the condition ν ≤ ν_0_ (i.e., the *coincidence* region delimited by the straight lines ν_0_ = ν—red— and ν_0_ = 10ν—blue— in Figure 7). It is appreciated that by neglecting many-ion correlations, the hopping frequency is slightly overestimated in average. In particular, 6 out of the 15 analyzed materials are represented by points that clearly lie on the outer region above the selected coincidence interval. For instance, a large frequency discrepancy amounting from 1 to 2 orders of magnitude are obtained for Li_10_GeS_2_P_12_, LiNbO_3_, Cu_2_Se, CuI, and AgI. On the other hand, the ν’s estimated for Li_7_La_3_Zr_2_O_12_, Li_2_SnS_3_, SrTiO_3_, and CsPbBr_3_, among others, agree fairly well with the approximate hopping frequencies obtained from the corresponding tracer diffusion coefficients.

For Li-, Cu- and Ag-based SSEs, the results enclosed in Figure 7 indicate that ν_0_, in general, is a not a good approximation for ν since the former overestimates the latter. Contrarily, the points obtained for halide-, Na-, and O-based SSEs, as well as for some Li-based SSEs, are located inside or very close to the selected coincidence region, meaning that ν_0_ is a reasonably good approximation for ν. Based on these findings, along with those presented in previous sections (Figure 4a), we can state that the hopping frequency of materials in which the correlations between mobile particles extend to many ions (only few ions) is likely to be poorly (fairly well) approximated by the tracer diffusion coefficient. This conclusion is quantitatively novel since failure of the relations obtained in the dilute-solution limit now can be directly associated with the average number of correlated mobile ions.

Finally, in order to quantify the influence of neglecting many-ion correlation on the calculation of the activation energy barrier for ionic migration, *E*_a_, we estimated this quantity for Li_7_La_3_Zr_2_O_12_ (LLZO) and Li_10_GeS_2_P_12_ (LGSP), considering both the tracer and full ionic diffusion coefficients (Computational Methods).^19−21^ We selected these two materials because the first lies inside the coincidence interval defined for the ion hopping frequency, while the second is outside. For LLZO, it was found that when disregarding many-ion correlations *E*_a_ amounted to 0.16 eV, whereas it decreased to 0.14 eV when accounting for them. For LGSP, we obtained similar results, in particular, 0.21 and 0.20 eV from the tracer and full ionic diffusion coefficients, respectively. Therefore, it may be concluded that the influence of neglecting many-ion correlations on the estimation of *E*_a_ appears to be less significant than that for ν.

## 4 Conclusions

In conclusion, we have carried out a comprehensive and unsupervised many mobile ion correlation analysis for several families of SSEs based on the k-means clustering approach, which has been implemented in the freely available open-source python code IonDiff.^31^ An exponential decaying law is found to correctly describe the general probability density distribution governing the degree of concertation between many mobile ions in SSEs. Accordingly, *n*-ion-coordinated diffusion processes with 2 < *n* are found to be more frequent than pairwise-coordinated diffusive events, although the latter hold the largest individual probability. For the particular case of Li-based SSEs, the average number of correlated mobile ions is estimated to be 10 ± 5, and interestingly, this result turns out to be practically independent of temperature. Furthermore, our data-driven analysis concludes that promising fast-ionic conductors characterized by large ion diffusion coefficients strongly and positively correlate with ample hopping lengths and long hopping times but not with high hopping frequencies and short interstitial residence times. Finally, it is shown that neglecting many-ion correlations generally leads to a modest overestimation of the hopping frequency that roughly is proportional to the average number of correlated mobile ions. Overall, our work leverages the fundamental understanding of ionic transport and SSEs and elaborates on the limitations of using formulas obtained in the dilute-solution approximation for describing technologically relevant fast-ionic conductors.

## 5 Computational Methods

### 5.1 First-Principles Calculation Outline

Ab initio calculations based on density functional theory (DFT)^39^ were performed to analyze the physicochemical properties of bulk SSEs. We performed these calculations with the VASP code^40^ by following the generalized gradient approximation to the exchange-correlation energy due to Perdew et al.^41^ For some halide compounds, likely dispersion interactions were captured with the D3 correction scheme developed by Grimme and co-workers.^42^ The projector augmented-wave method was used to represent the ionic cores,^43^ and, for each element, the maximum possible number of valence electronic states was considered. Wave functions were represented in a plane-wave basis typically truncated at 750 eV. By use of these parameters and dense **k**-point grids for Brillouin zone integration, the resulting zero-temperature energies were converged to within 1 meV per formula unit. In the geometry relaxations, a tolerance of 0.005 eV·Å^–1^ was imposed on the atomic forces.

### 5.2 First-Principles Molecular Dynamics Simulations

AIMD simulations based on DFT were performed in the canonical (*N*, *V*, *T*) ensemble (i.e., constant number of particles, volume, and temperature) for all the analyzed materials. The selected volumes were those that were determined at zero temperature; hence, thermal expansion effects were neglected. Nevertheless, based on previously reported molecular dynamics tests,^8^ thermal expansion effects are not expected to affect significantly the estimation of ion-transport features at moderate temperatures. The concentration of ion vacancies in the nonstoichiometric compounds was also considered independent of the temperature and equal to ∼1–2%. The temperature in the AIMD simulations was kept fluctuating around a set-point value by using Nose–Hoover thermostats. Large simulation boxes containing *N* ∼ 200–300 atoms were employed in all the cases, and periodic boundary conditions were applied along the three supercell vector directions. Newton’s equations of motion were integrated by using the customary Verlet’s algorithm, and a time-step length of δ*t* = 1.5 × 10^–3^ ps. Γ-Point sampling for integration within the first Brillouin zone was employed in all the AIMD simulations.

Our finite-temperature simulations typically comprised long simulation times of *t*_total_ ∼ 100 ps. The first ∼25 ps of the AIMD simulations correspond to the system equilibration and hence were disregarded in the subsequent ion diffusion analysis. For each material, we typically ran an average of 3 AIMD simulations at different temperatures within the range 300 ≤ *T* ≤ 1200 K, considering both stoichiometric and nonstoichiometric compositions.^24^ Previous tests performed on the numerical bias stemming from the finite size of the simulation cell and duration of the molecular dynamics runs reported in previous work^8^ indicate that the adopted *N* and *t*_total_ values should provide reasonably well converged results for the ion diffusivity and vibrational density of states of SSEs. A systematic study of how finite size effects (i.e., supercell size and AIMD simulation duration) may affect our many-ion correlation results is presented in the Supporting Discussion.

The MSD was estimated with the formula
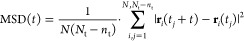
3where **r**_*i*_(*t*_*j*_) represents the position of the mobile ion *i* at time *t*_*j*_ (=*j* · δ*t*), *t* is the lag time, *n*_t_ = *t*/δ*t*, *N* is the total number of mobile ions, and *N*_t_ is the total number of time steps (equivalent to ∼100 ps). The maximum *n*_t_ was chosen equal to *N*_t_/2 (equivalent to ∼50 ps) in order to accumulate enough statistics to significantly reduce the MSD(*t*) fluctuations at large *t*’s. The tracer diffusion coefficient, *D*, then was obtained based on the Einstein relation
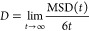
4In practice, we considered 0 < *t* ≤ 50 ps and estimated *D* by performing linear fits to the averaged MSD(*t*) obtained over the last 25 ps. When taking into account many-ion correlations, the full diffusion coefficient was estimated by considering additional *i*–*j* particle positions crossed terms in eq 3.^21^

### 5.3 Spatiotemporal Correlation Function

The van Hove correlation function, *G*(Δ*r*, Δ*t*), provides information on the spatiotemporal distribution of particles during a simulation. This two-dimensional function can be intuitively divided into a “self”, *G*_s_, and a “distinct”, *G*_d_, part. The former describes the displacements of a specific particle throughout time, while the latter describes the relations of a particle with the rest, namely
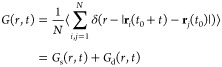
5where **r** represents the atomic position, indices *i* and *j* are runs over all the mobile particles, δ(*x*) is the Dirac delta function, *t*_0_ is an arbitrary time, and averages are estimated over the total simulation time. The “self” and “distinct” parts of the van Hove correlation function are then defined as
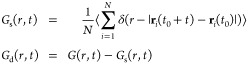
6

### 5.4 IonDiff Software

The freely available open-source python code IonDiff^31^ is based on an unsupervised k-means clustering algorithm (see the next section for additional details). By fully taking into account periodic boundary conditions, IonDiff assigns a spatial point (i.e., center of vibration) to every particle in the simulation supercell at each simulated time step. The centers of vibration then are compared with the stoichiometric equilibrium lattice so that (1) ion-hopping events can be straightforwardly identified without the need of defining any arbitrary length or parameter, and (2) metastable positions can be also readily determined. The residence time for a particular metastable position is estimated as the number of simulation steps associated with that location averaged over all of the particles. The only required input files are two: (1) one containing the positions of the particles throughout the whole simulation (e.g., the XDATCAR file in the case of VASP calculations) and (2) another detailing the length and number of time steps (e.g., the INCAR file in the case of VASP calculations).

### 5.5 K-Means Clustering

The unsupervised algorithm devoted to identifying diffusive particles and their respective paths in molecular dynamics simulations is based on the k-means clustering approach. The implementation of the k-means clustering algorithm in the Scikit-learn python package^45^ was used in practice. The number of clusters at each time step, *K*, was selected based on the average silhouette method. In particular, the chosen *K* corresponds to that which maximizes its average value over all possible 2 ≤ *K* cases (see main text). An arbitrary but reasonable confidence threshold value of 0.7 was imposed for the silhouette coefficient *S* (eq 7). This means that if the maximum average silhouette coefficient amounted to less than 0.7, then the condition *K* = 1 was automatically imposed.

*M*_*I*_ being the number of points in cluster *I*, with *M*_*I*_ > 1, the silhouette coefficient for a data point in that cluster, *i*, is mathematically defined as
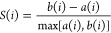
7where
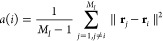
8
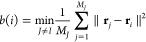
9By proceeding in this manner, the similarity of a point within its own cluster and its dissimilarity with the others were simultaneously optimized.

## Supporting Information Available

Detailed description of the systems simulated with the AIMD technique, metastable site identification analysis, temperature dependence of the ion hopping frequency, temperature dependence of the distance for the materials analyzed in this study, discussion on the use of graph-based machine-learning force fields for extending the number of analyzed SSEs, and how size effects may affect the many-ion correlation results presented in this study (PDF)
