# Supplementary material for: How Concerted Are Ionic Hops in Inorganic Solid-State Electrolytes?
Source: J Am Chem Soc. 2024 Mar 18;146(12):8269–79. doi: 10.1021/jacs.3c13279 (PMC11583204; doi:10.1021/jacs.3c13279)
Supplement: Supplementary file 1 — ja3c13279_si_001.pdf [file ja3c13279_si_001.pdf]

Supporting Information for "How concerted are ionic hops in inorganic solid-state electrolytes?"

Cibrán López

Departament de Física, Universitat Politècnica de Catalunya, 08034 Barcelona, Spain

Barcelona Research Center in Multiscale Science and Engineering,

Universitat Politècnica de Catalunya, 08019 Barcelona, Spain and

Institut de Ciència de Materials de Barcelona,

ICMAB–CSIC, Campus UAB, 08193 Bellaterra, Spain

Riccardo Rurali

Institut de Ciència de Materials de Barcelona,

ICMAB–CSIC, Campus UAB, 08193 Bellaterra, Spain

Claudio Cazorla*

Departament de Física, Universitat Politècnica de Catalunya, 08034 Barcelona, Spain and

Barcelona Research Center in Multiscale Science and Engineering,

Universitat Politècnica de Catalunya, 08019 Barcelona, Spain

Composition	Space group	Number of atoms	Temperatures (K) [D (Å^2/ps)]
$Li_{10}GeS_2P_{12}$	$P4_2mc$	200	650, 900, 1150, 1400 [0.16,0.364,0.6273,0.96]
$Li_2B_{12}H_{12}$	Pa_3	832	600 [0.01453]
$Li_{1.94}B_{12}H_{12}$	Pa_3	830	600 [0.02067]
$Li_{1.97}SnS_3$	$C2/c$	382	800, 1000, 1200 [0.00082,0.003253,0.00212]
$Li_3La_4Ti_8O_{24}$	$Pmmm$	312	700, 900, 1100 [0.003513,0.02153,0.037]
Li_3N	$P6/mmm$	256	500, 700, 900, 1100 [0.0047267,0.0594,0.154,0.2733]
$Li_{2.95}O_{0.98}Cl_{0.98}$	$P4/mmm$	315	1000, 1250, 1500, 1650 [0.005633,0.028667,0.54,1.026667]
$Li_7La_3Zr_2O_{12}$	$I4_1/acd$	384	800 [0.0035133]
$Li_{6.88}La_3Zr_2O_{12}$	$I4_1/acd$	382	400, 600, 800 [0.0039733,0.01033,0.0244]
$Li_7P_3S_{11}$	$P\bar{1}$	336	400, 600, 800 [0.032733,0.194,0.41]
$Li_{6.94}P_3S_{11}$	$P\bar{1}$	335	600, 800 [0.2033,0.442]
$Li_{0.97}Ga_{0.97}O_{1.94}$	$Pna2_1$	124	500, 700, 900, 1100, 1300, 1500 [0.0002133,0.000506,0,0.0017467,0.00664,0.0072]

(Continuation)			
Composition	Space group	Number of atoms	Temperatures (K) [D (Å^2/fs)]
$LiIO_3$	$P6_3$	270	1100 [0.000062]
$LiI_{0.94}O_3$	$P6_3$	267	300, 500, 700, 900 [0.0015933,0.00041866,0.008867,0.0116]
$Li_{0.94}Mn(HCO_2)_3$	$P2_13$	223	500 [0.000036067]
$Li_{0.97}NbO_3$	$R3c$	179	700, 900 [0.00146,0.00867]
$LiTi_2(PO_4)_3$	$P\overline{3}c1$	432	400, 600 [0.0008,0.00045867]
$Li_{0.92}Ti_2(PO_4)_3$	$P\overline{3}c1$	430	400, 600, 800 [0.0004573,0.0004353,0]

Supporting Table I: Li-based solid-state electrolytes (SSE) considered in this study for which DFT-AIMD finite-temperature simulations were performed. Ion diffusion coefficients, D, listed as "0" are $< 10^{-5}$ Å^2/ps.

Composition	Space group	Number of atoms	Temperatures (K) [D (Å^2/ps)]
Na_3SbS_4	$I\bar{4}3m$	288	700, 900 [0,0.0000036]
$Na_{0.94}Mn(HCO_2)_3$	$P2_13$	223	500 [0.0009]
AgI	$F\bar{4}3m$	250	200, 350, 500 [0.0673,0.1086,0.2586]
Ag_3SBr	$Pm\bar{3}m$	320	600 [0.03]
Ag_3SI	$Pm\bar{3}m$	320	600 [0.049]
$Cu_{1.99}Se$	$Fm\bar{3}m$	215	300, 500, 700 [0.003006,0.04173,0.192]
CuI	$F\bar{4}3m$	216	500, 700 [0.04946,0.1853]
Na_3P_{11}	$Pbcn$	448	1000 [0.010431]
$Na_{2.98}P_{11.02}$	$Pbcn$	447	1000 [0.00371]

Supporting Table II: Na-, Ag- and Cu-based SSE considered in this study for which DFT-AIMD finite-temperature simulations were performed. Ion diffusion coefficients, D, listed as "0" are $< 10^{-5}$ Å^2/ps.

Composition	Space group	Number of atoms	Temperatures (K) [D (Å^2/ps)]
BaF_2	$Fm\bar{3}m$	192	1000, 1375, 1500 [0,0.27267,0.673]
CaF_2	$Fm\bar{3}m$	192	1250, 1500 [0.0026067,0.2253]
$CsPbBr_3$	$Pm\bar{3}m$	180	500, 700, 850 [0.00986,0.0016467,0.038067]
$CsPbBr_{2.97}$	$Pm\bar{3}m$	179	500, 700, 900 [0.0043,0.0104,0.02053]
$SrCl_2$	$C2/m$	192	2000, 2500 [0.0022067,0.5293]
SrF_2	$Fm\bar{3}m$	192	1500, 1750 [0.1193,0.5967]
Bi_2O_3	$Pn\bar{3}m$	180	500 [0.002053]
$SrCoO_{2.75}$	$Pbam$	152	200, 400 [0.0021867,0.00188]
$SrTiO_{2.95}$	Pc	317	1500, 2000 [0.00102,0.0030067]

Supporting Table III: Halide- and O-based SSE considered in this study for which DFT-AIMD finite-temperature simulations were performed. Ion diffusion coefficients, D, listed as "0" are $< 10^{-5}$ Å^2/ps.

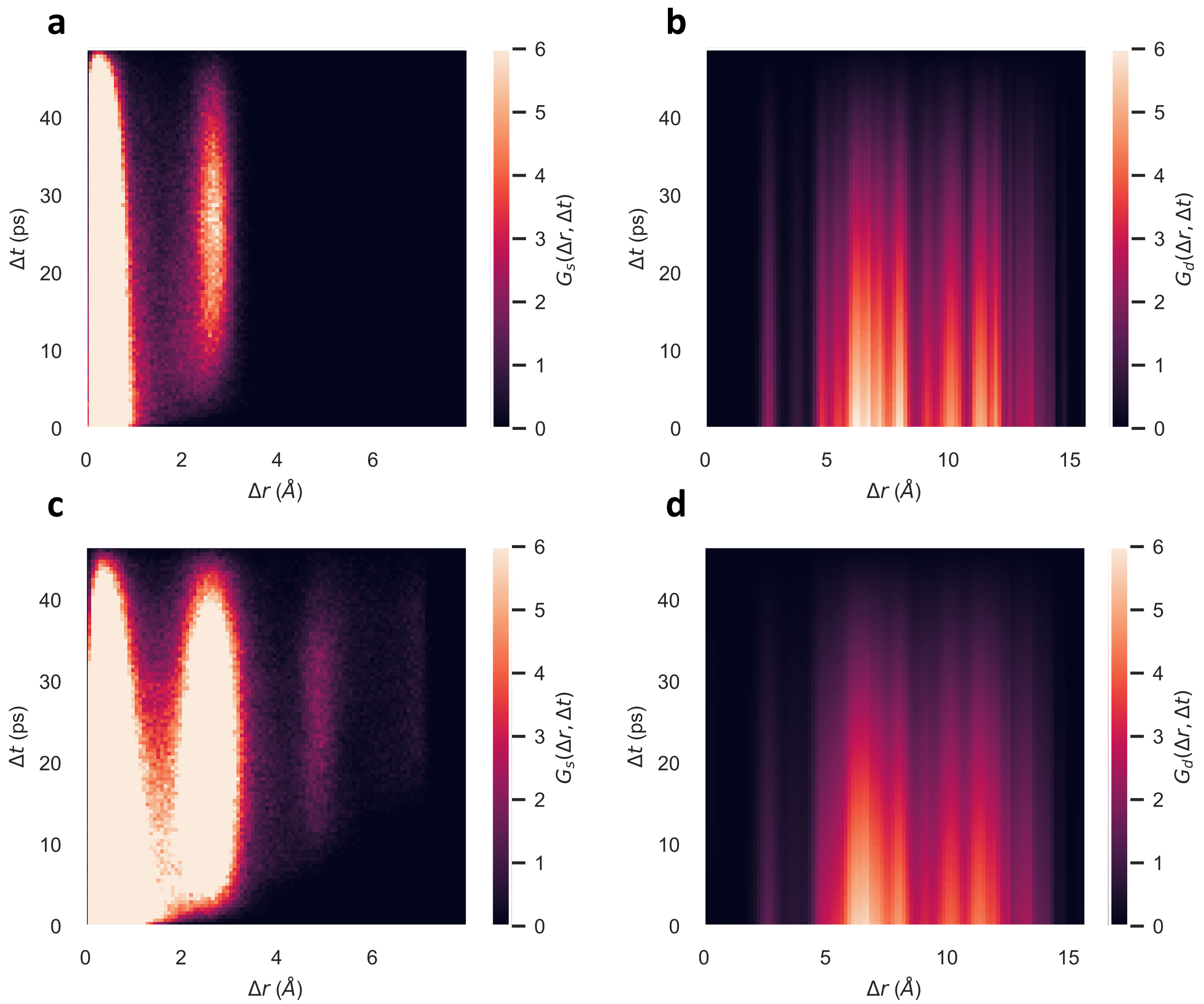

Supporting Figure 1: (a)–(c) Self and (b)–(d) distinct parts of the van Hove correlation function estimated for $Li_7La_3Zr_2O_{12}$ (LLZO) at (a)–(b) $T = 400$ K and (c)–(d) $T = 800$ K.

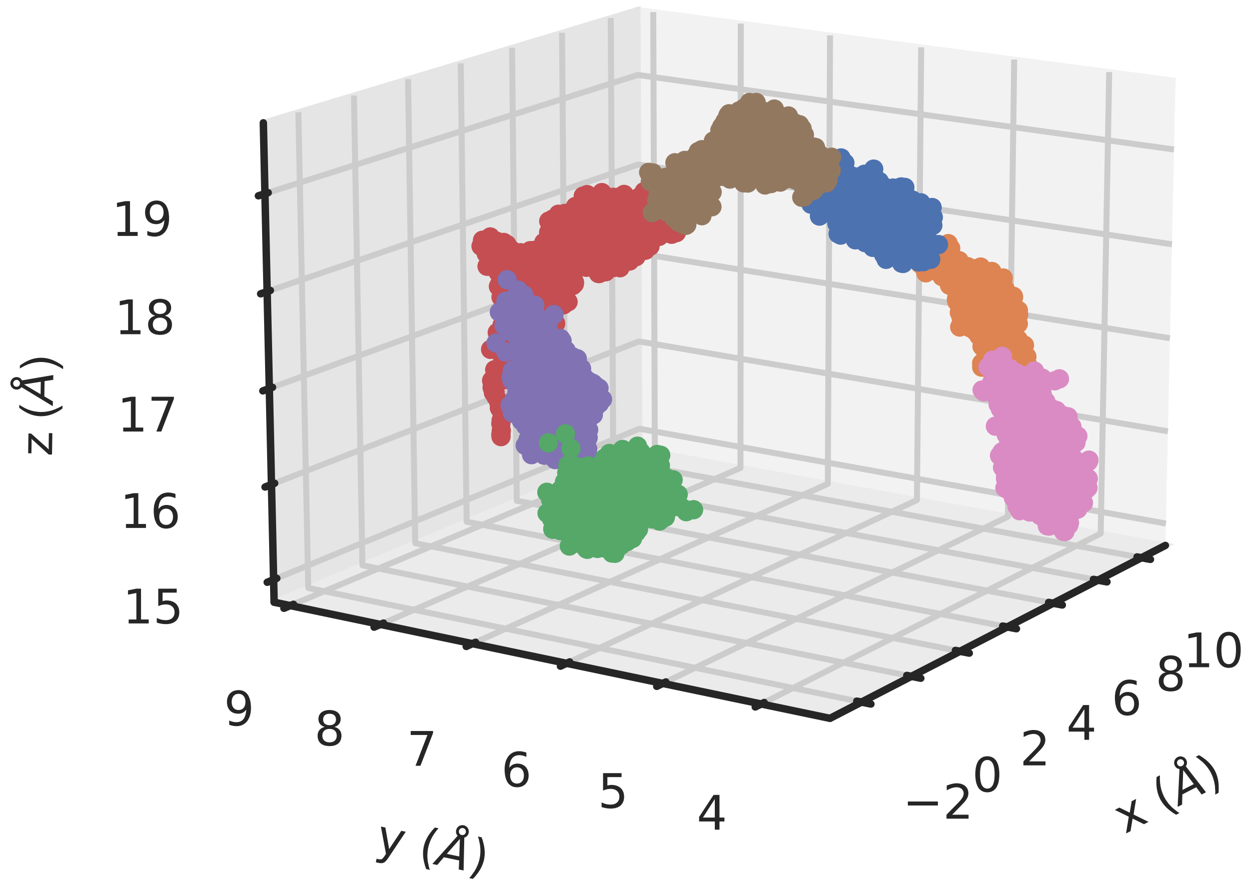

Supporting Figure 2: Example of a sequence of ionic hops, n, identified with the IonDiff software during a same simulation. It is clearly appreciated that $n > 2$, as indicated by the different colors employed in the representation.

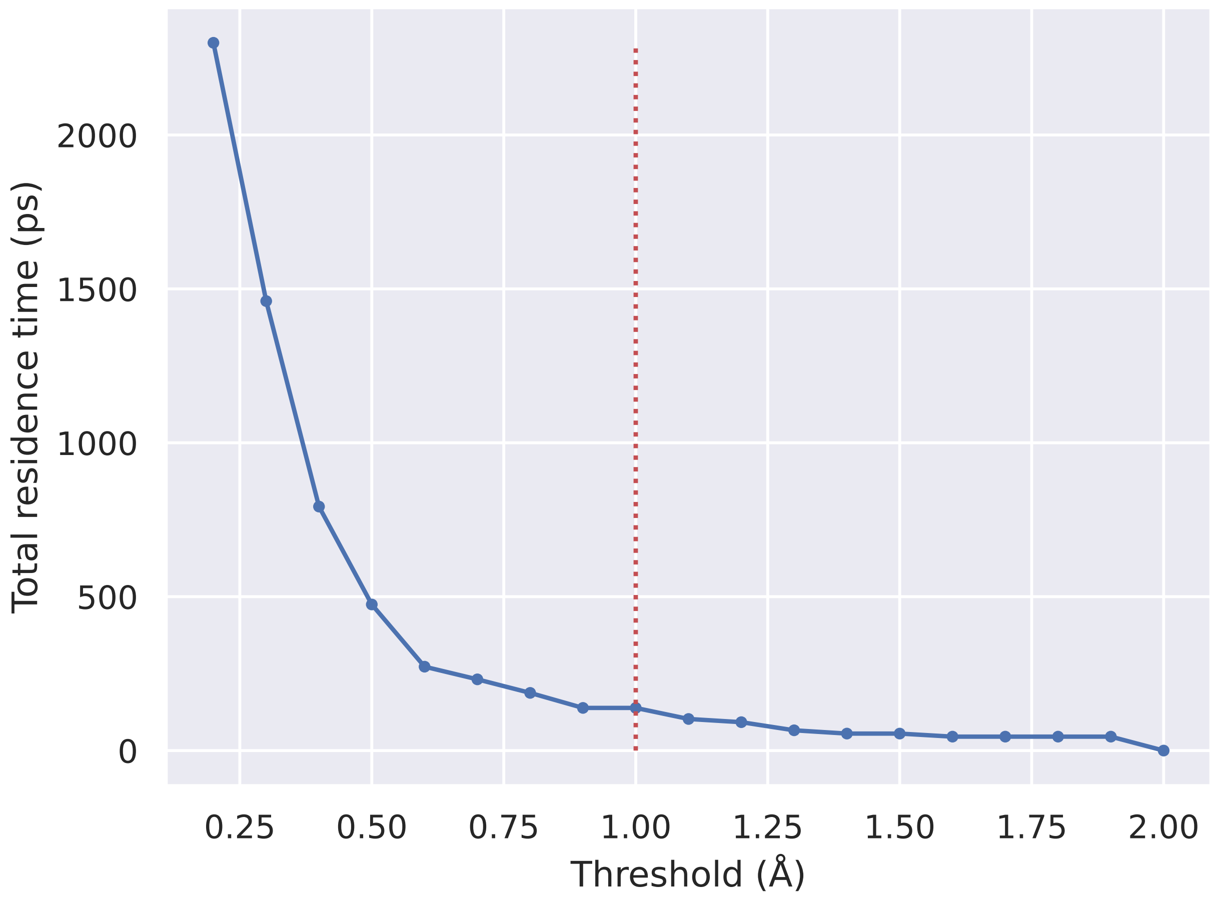

Supporting Figure 3: Selection of the threshold distance employed to distinguish among metastable and equilibrium vibrational centers. The enclosed simulation results were obtained for $Li_7La_3Zr_2O_{12}$ (LLZO) at $T = 800$ K; additional tests were carried out for other materials (e.g., $AgCrSe_2$) leading to analogous conclusions.

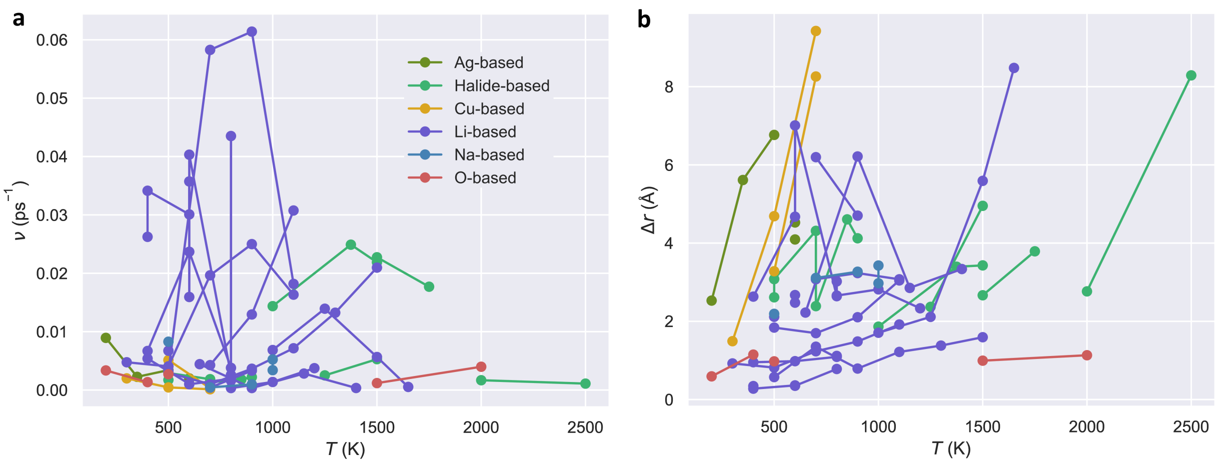

Supporting Figure 4: Temperature dependence of the (a) ion hopping frequency and (b) ion hopping distance estimated for all the materials considered in this study.

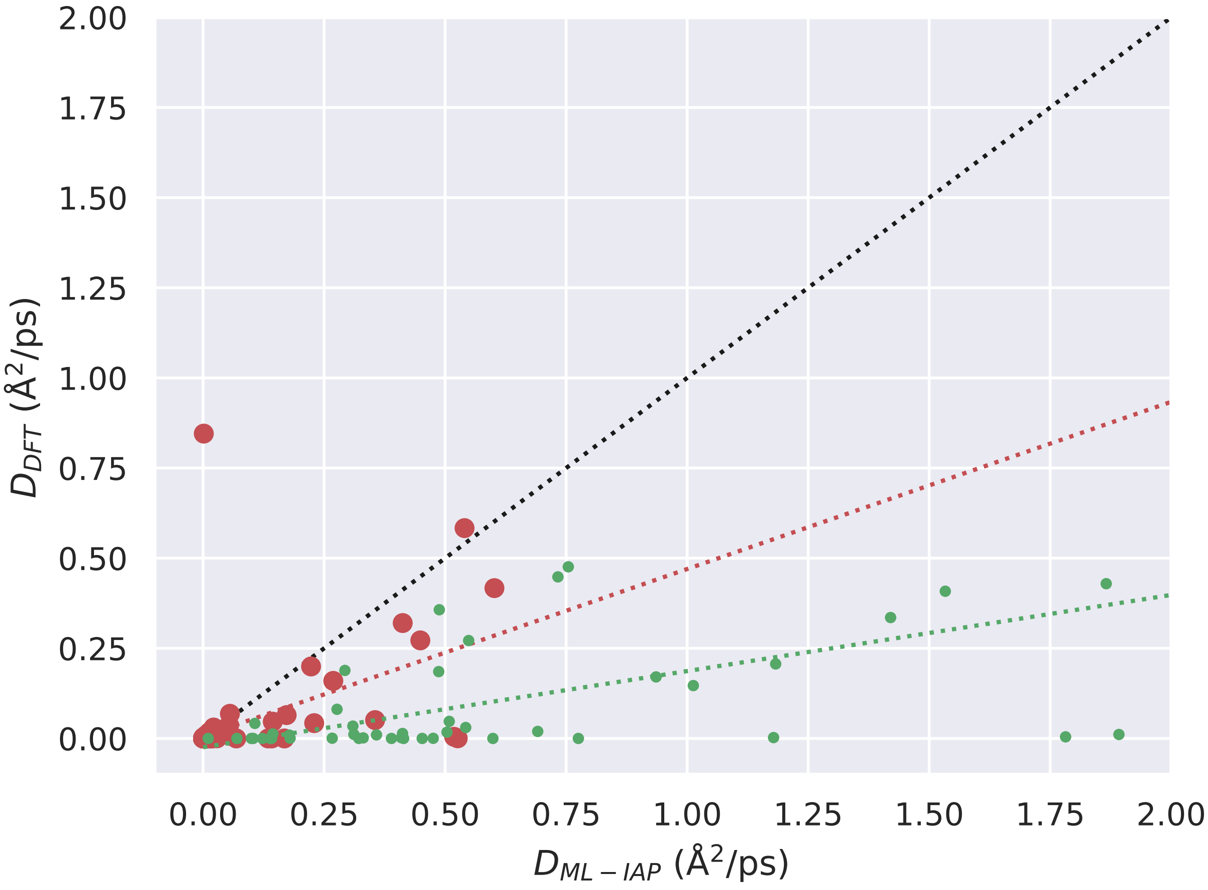

Supporting Figure 5: Comparison of the ionic diffusion coefficients, D, estimated with first-principles AIMD methods[1,2], labelled "DFT", and with the universal graph-based force field M3GNet[4], labelled "ML-IAP". The results summarize a total of 166 full simulations that involved different materials and temperatures. In both DFT and ML-IAP modalities, the adopted technical parameters (i.e., supercell sizes and total simulation times) were identical. The red dots, which sum up to a total of 119, correspond to well-behaved DFT and ML-IAP simulations in which the simulated crystals did not melt. The green dots, which sum up to a total of 47, correspond to ML-IAP simulations in which the crystals melted whereas in the DFT simulations remained stable. The red (green) dotted line represents a linear fit to the D results represented by red (green) dots. The mean absolute error (MAE) associated with the results represented by red (green) dots is of 0.034 Å^2 ps^{-1} (0.544 Å^2 ps^{-1}). The black dotted line represents the ideal case in which the DFT and ML-IAP results were identical.

SUPPORTING DISCUSSION

We performed two different sets of tests aimed at assessing (i) the performance of affordable machine learning force fields in reproducing the AIMD database of fast-ion conductors[1,2] that has been analyzed in this study with the IonDiff software[3], and (ii) the impact of finite-size effects on the many-ion correlation conclusions presented in the main text. For these tests, we employed the as-delivered universal graph-based force field M3GNet[4] (i.e., without performing any additional specific training on it).

Supporting Figure 5 shows the results of the first set of tests directed at exploring the performance of computationally affordable machine learning force fields[4] in reproducing the main traits of the analysed AIMD SSE database[1,2]. Specifically, we compare the ionic diffusion coefficients, D, estimated with first-principles AIMD methods, "DFT", and M3GNet[4], "ML-IAP", considering exactly the same supercell sizes and total simulation times in both types of calculations. The results comprise a total of 166 full molecular dynamics simulations involving different materials and temperatures[1,2]. The red dots in the Supporting Figure 5, which sum up to a total of 119, correspond to qualitatively consistent ML-IAP and DFT simulations in which the simulated crystals did not melt. The green dots, which sum up to a total of 47, correspond to qualitatively inconsistent ML-IAP and DFT simulations in which the crystals melted in the ML-IAP simulations while remained stable in the DFT simulations. The red (green) dotted line in the Supporting Figure 5 corresponds to a linear fit to the D results represented by red (green) dots. It is clearly appreciated that the employed machine learning force field tends to significantly overestimate the ionic diffusion coefficients calculated with accurate first-principles methods. In particular, the mean absolute error (MAE) associated with the red ML-IAP D results amounts to 0.034 Å^2 ps^{-1} ($3.4 \cdot 10^{-6}$ cm^2 s^{-1}), which turns out to be quite sizeable. In addition, the stability of the crystal phases appears to be acutely compromised in the ML-IAP simulations (green dots). Thus, in view of our results, the use of generic machine learning force fields that have not undergone any specific training on SSE is strongly discouraged for SSE modelling.

The second set of tests were directed at evaluating the impact of finite-size effects on the many-ion correlation results reported in the main text. Particularly, we evaluated changes in the average number of mobile correlated ions, N_{corr}, upon substantially increasing either the total number of ions or the total duration of the simulations. These tests were performed with

the universal graph-based force field M3GNet[4] for $Li_7La_3Zr_2O_{12}$ (LLZO) and $Li_{10}GeS_2P_{12}$ (LGSP) at $T = 400$ K (at higher temperatures, the simulated materials either melted or exhibited unrealistically large Li diffusion coefficients thus conditions $T \geq 400$ K were disregarded). The results obtained for both LLZO and LGSP were averaged in order to improve the statistics. The technical parameters of the reference simulations were $N_{\rm ion} = 382$ for LLZO, $N_{\rm ion} = 200$ for LGSP and $t_{\rm total} = 100$ ps for both systems. Subsequently, $N_{\rm ion}$ was increased to $1,582$ for LLZO and to $1,600$ for LGSP while keeping a total simulation time of 100 ps. Next, $t_{\rm total}$ was increased up to $1,000$ ps while keeping the number of ions to 382 for LLZO and to 200 for LGSP. The reference average $N_{\rm corr}$ value estimated with the IonDiff software[3] was equal to 4 ± 2, which turns out to be consistent within the statistical uncertainties with, although is appreciably smaller than, the average value determined from the AIMD simulations considering many Li-based SSE (that is, 10 ± 5). By constraining the total number of ions to 382 and 200 and substantially increasing the total simulation time the estimated $N_{\rm corr}$ amounted to 11 ± 6, which is larger than, but still compatible within the statistical uncertainties, with the reference $N_{\rm corr}$ value. On the other hand, when constraining the total simulation time to 100 ps and substantially increasing the total number of ions to $1,582$ and $1,600$, the estimated $N_{\rm corr}$ amounted to 5 ± 4, which is pretty similar to the reference $N_{\rm corr}$ value. Thus, overall, it may be concluded that despite size-effects are not negligible the presented many-ion correlation conclusions appear to be robust.

SUPPORTING REFERENCES

* Electronic address: claudio.cazorla@upc.edu

1 López, C., Emperador, A., Saucedo, E., Rurali, R. and Cazorla, C. Universal ion-transport descriptors and classes of inorganic solid-state electrolytes. *Mater. Horiz.* **10**, 1757 (2023).

2 The DFT-AIMD database analyzed in this work can be found at the URL: https://superionic.upc.edu/

3 López, C., Rurali, R. and Cazorla, C. https://github.com/IonRepo/IonDiff

4 Chen, C. and Ong, S.-P. A universal graph deep learning interatomic potential for the periodic table. *Nat. Comput. Sci.* **2**, 718 (2022).
